# Molecular characterization of the viral structural protein genes in the first outbreak of dengue virus type 2 in Hunan Province, inland China in 2018

**DOI:** 10.1186/s12879-021-05823-3

**Published:** 2021-02-10

**Authors:** Jiaoqiong Guan, Zhanlong He, Meng Qin, Xialin Deng, Jun Chen, Suqin Duan, Xiaojun Gao, Yue Pan, Junying Chen, Yaping Yang, Shijun Feng, Qiangming Sun

**Affiliations:** 1 Institute of Medical Biology, Chinese Academy of Medical Sciences, and Peking Union Medical College, Kunming, People’s Republic of China; 2 Yunnan Key Laboratory of Vaccine Research and Development on Severe Infectious Diseases, Kunming, People’s Republic of China; 3 Yunnan Key Laboratory of Vector-borne Infectious Disease, Kunming, People’s Republic of China; 4 Beijing Advanced Innovation Center for Soft Matter Science and Engineering, College of Life Science and Technology, Beijing University of Chemical Technology, Beijing, People’s Republic of China; 5 Qiyang People’s Hospital, Yongzhou, Hunan People’s Republic of China

**Keywords:** Dengue virus, Structural protein, Phylogenetic trees, Protein structure, Selection pressure

## Abstract

**Background:**

An unexpected dengue outbreak occurred in Hunan Province in 2018. This was the first dengue outbreak in this area of inland China, and 172 cases were reported.

**Methods:**

To verify the causative agent of this outbreak and characterise the viral genes, the genes encoding the structural proteins C/prM/E of viruses isolated from local residents were sequenced followed by mutation and phylogenetic analysis. Recombination, selection pressure, potential secondary structure and three-dimensional structure analyses were also performed.

**Results:**

Phylogenetic analysis revealed that all epidemic strains were of the cosmopolitan DENV-2 genotype and were most closely related to the Zhejiang strain (MH010629, 2017) and then the Malaysia strain (KJ806803, 2013). Compared with the sequence of DENV-2SS, 151 base substitutions were found in the sequences of 89 isolates; these substitutions resulted in 20 non-synonymous mutations, of which 17 mutations existed in all samples (two in the capsid protein, six in the prM/M proteins, and nine in the envelope proteins). Moreover, amino acid substitutions at the 602nd (E322:Q → H) and 670th (E390: N → S) amino acids may have enhanced the virulence of the epidemic strains. One new DNA binding site and five new protein binding sites were observed. Two polynucleotide binding sites and seven protein binding sites were lost in the epidemic strains compared with DENV-2SS. Meanwhile, five changes were found in helical regions. Minor changes were observed in helical transmembrane and disordered regions. The 429th amino acid of the E protein switched from a histamine (positively charged) to an asparagine (neutral) in all 89 isolated strains. No recombination events or positive selection pressure sites were observed. To our knowledge, this study is the first to analyse the genetic characteristics of epidemic strains in the first dengue outbreak in Hunan Province in inland China.

**Conclusions:**

The causative agent is likely to come from Zhejiang Province, a neighbouring province where dengue fever broke out in 2017. This study may help clarify the intrinsic geographical relatedness of DENV-2 and contribute to further research on pathogenicity and vaccine development.

**Supplementary Information:**

The online version contains supplementary material available at 10.1186/s12879-021-05823-3.

## Background

Dengue fever (DF), an ancient disease with a history of approximately 2000 years, is caused by four different but closely related dengue viruses (DENV-1, DENV-2, DENV-3, and DENV-4) and is mainly transmitted by female *Aedes aegypti* or female *Aedes albopictus* [1, 2]. DF occurs in tropical and subtropical urban and semi-urban areas around the world. The global dengue epidemic has spread quickly in recent decades from 9 endemic countries before 1970 to 128 in 2012 [3]. The incidence of dengue has also increased dramatically from 1.2 million in 2008 to 3.9 million in 2015 [4, 5]. In 2016, there were more than 2.38 million cases in the Americas alone, of which Brazil accounted for approximately 1.5 million cases. Meanwhile, more than 375,000 cases were reported in the Western Pacific region, including 176,000 cases in the Philippines and 100,000 cases in Malaysia [6]. However, the number of dengue cases reported in the Americas in 2017 was 580,000, which was approximately 78.9% fewer than the previous year. According to data provided by the World Health Organization (WHO), the number of cases in the first quarter of 2018 decreased by 27% compared with that in the same period of 2017; during this period, DF cases were mainly reported in countries such as Paraguay, Argentina, Bangladesh, Cambodia, India, Myanmar, Malaysia, Pakistan, Thailand, Yemen, and China and were mainly caused by the DENV-1 and DENV-2 serotypes [7].

DF has become a serious public health problem in China. According to data provided by the Chinese Center for Disease Control (CCDC), 757,243 people have been infected in the past 42 years [8, 9], and these infections largely occurred in Hainan [10], Guangdong [11, 12], Zhejiang [13, 14], Fujian [15], Taiwan, and Yunnan [16–19]. In 2018, an unexpected dengue outbreak occurred for the first time in Hunan Province, an inland province of China. According to data provided by the Centers for Disease Control and Prevention (CDC), the earliest DF case was reported on September 2. On October 6, 172 infected individuals were confirmed as NS1-positive, with one death in Hunan Province. Qiyang County was the location most seriously affected by the epidemic; 73 cases were confirmed in Qiyang County from September 8 to September 14, accounting for 76.04% of the total confirmed cases in this area. The ratio of female to male infected patients was 1.04 to 1 (49:47), with an average age of 49.5 years (ranging from 11 to 84 years old). It should be noted that no dengue cases were found in this area from 2000 to 2013; only five imported cases were reported between 2014 and 2017, and no local cases were reported in Qiyang County.

This was the first dengue outbreak in Hunan, an interior province of China. This outbreak provides us with an early warning that dengue fever has gradually spread inland from China’s coastal and border regions and highlights the urgent need to monitor the cross-border and cross-regional spread of dengue virus. The purpose of this study was to verify the causative agent and analyse the molecular characteristics of the epidemic strain in this outbreak.

## Methods

### The geographic analysis of Hunan Province and study design

The geographical distribution map of dengue fever in China over the years was created by Chinese mapping and drawing software. Blood samples of patients were collected from two local hospitals responsible for the treatment of DENV patients (Qiyang People’s Hospital and the Nongshan Hospital) during the 2018 dengue outbreak. The dengue fever epidemic situation in the surrounding areas of Hunan Province was also included in this analysis.

### Dengue virus NS1 antigen detection

The DENV NS1 antigen was detected by the colloidal gold method according to the manufacturer’s instructions (Dengue NS1/IgG/IgM Test Cassette, Guangzhou Biological Products, Guangzhou, China). When there were red bands in both the quality control area and the sample area, the results were considered positive.

### Viral RNA extraction, dengue virus identification and sequencing of structural protein genes

Viral RNA was extracted from 140 μl of patient serum using the QIAamp Viral RNA Mini Kit (Qiagen, Hilden, Germany; No. 52906) and then reverse transcribed into cDNA using the PrimeScriptTM II 1st Strand cDNA Synthesis Kit (Takara Bio, Shiga, Japan; No. 6210A). Universal primers for dengue virus and the specific primers for the four serotypes (Table S1) were used for polymerase chain reaction (PCR), and the serotypes were identified. The PCR conditions were as follows: denaturation at 95 °C for 5 min; 30 cycles of denaturation at 95 °C for 30 s, annealing at 55 °C for 30 s, and elongation at 72 °C for 30 s; and a final elongation step at 72 °C for 7 min. The PCR products were confirmed by agarose gel electrophoresis and sequenced at Sangon Biotech Co., Ltd. (Shanghai, China). Both forward and reverse sequencing were performed.

### Primer design

A total of three synthetic oligonucleotide primer pairs, F1/R1, F2/R2, and F3/R3 (Table S2), were designed to amplify overlapping fragments 2325 nucleotides in length that spanned the entire sequence encoding the structural proteins of DENV-2. All primers were designed using SnapGene software (version 3.2.1) based on the reference strain (GenBank Accession No. M29095). All primers were synthesized and purified by Sangon Biotech Co., Ltd.

### Molecular characteristics analysis

A total of 89 nucleotide sequences were assembled using BioEdit 7.1.3 (http://www.mbio.ncsu.edu/bioedit/bioedit.html) and then uploaded to the National Center for Biotechnology Information (NCBI) GenBank® database (http://www.ncbi.nim.nih.gov/GenBank/index.html) (GenBank IDs: MK543451–MK543470, MK543472–MK543478, MK543480–MK543492, and MK949396–MK949438) by the Sequin tool (version 15.50). Next, the mutations in the nucleotide sequences and translated amino acid sequences of the structural proteins of these 89 strains were analysed with BioEdit and Molecular Evolutionary Genetics Analysis (MEGA) software version 7.0. The secondary structures of the structural proteins were predicted by the Predict Protein Server (https://www.predictprotein.org/) for both epidemic and reference strains.

### Phylogenetic analysis

The sequences of the structural protein genes (C/prM/E) from the 89 epidemic strains were aligned by MEGA 7.0 and compared with those of 133 DENV reference strains, including four serotypes of standard strains (Table S3), which were collected from websites (https://www.viprbrc.org). Phylogenetic analysis was performed using MEGA 7.0 through the ML phylogeny test with a bootstrap of 1000 replications.

### Recombination and selection pressure analysis

For detection of recombination, the Genetic Algorithm (GARD) [20] online server of Datamonkey [21] was used for automatic analysis of reorganization events of the structural protein genes of the 111 DENV-2 reference sequences shown in Table S4 and the 89 epidemic strains in our study. The phylogenies server was used for analysis of selection pressure. In this research, the following four methods were adopted to estimate the locus-specific selection pressure: the Fixed Effect Likelihood (FEL) method [22], the Internal Fixed Effect Likelihood (IFEL) method [23], the Mixed Effect Evolution Model (MEEM) method [24], and the Fast, Unconstrained Bayesian AppRoximation (FUBAR) [25] method. If at least three of the four methods meet the requirement of ω > 1(ω = β/α) and have a *p*-value < 0.1 or a posterior prob. (α < β) > 0.9, then the positive selection of this site can be inferred.

## Results

### The geographic analysis of Hunan Province and study design

**Fig. 1:**
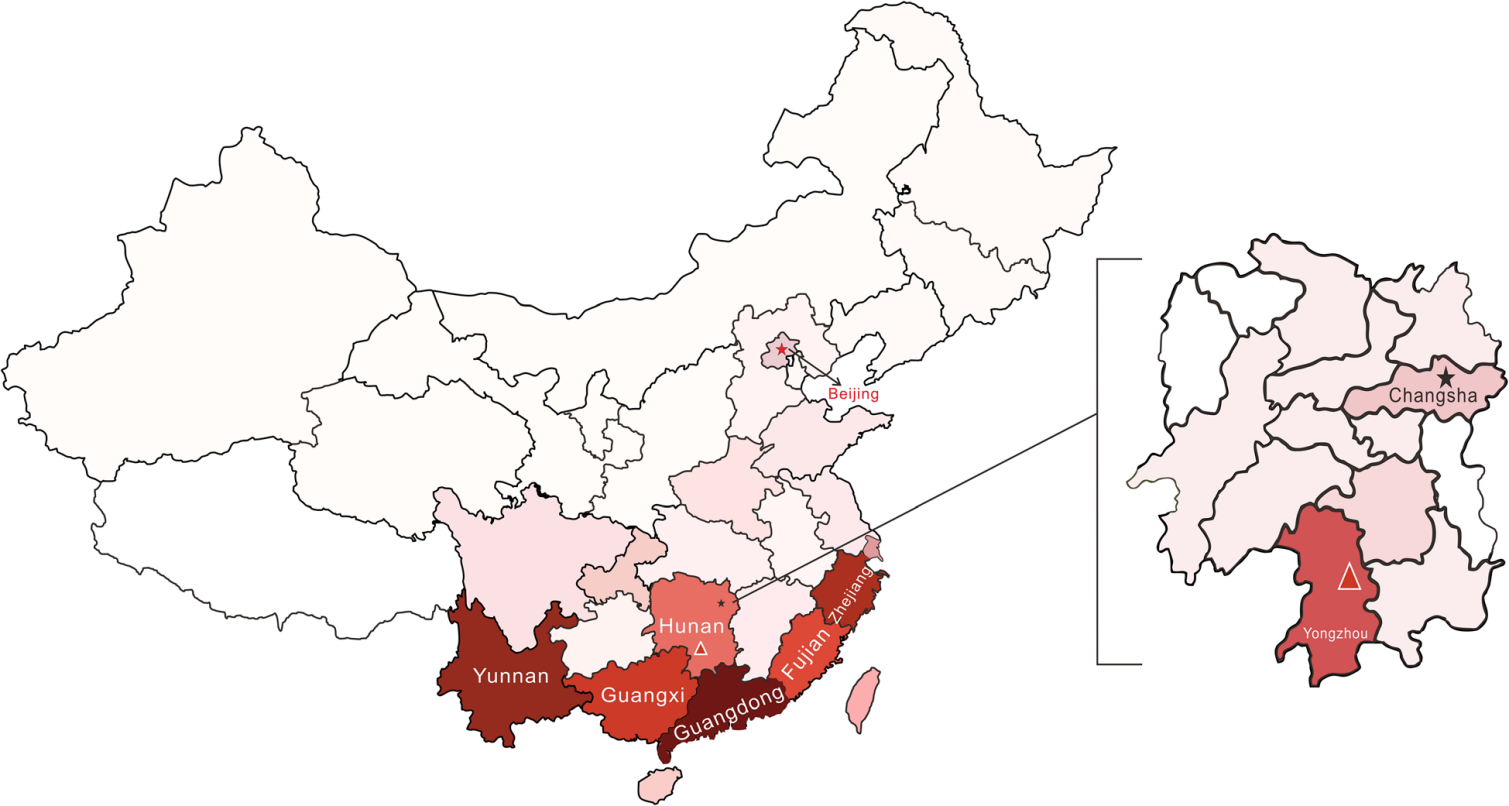
Geographic relationships between Hunan Province and other dengue outbreak areas in China. The intensity of the colour in the figure indicates the number of dengue cases; the darker the colour is, the more dengue cases there were in the area. The map of China shows the distribution of dengue cases in China in the past 15 years (from 2004 to 2018), and the map of Hunan Province shows the distribution of dengue cases in 2018. As visible in the figure, Hunan Province is surrounded by areas with a high incidence of dengue fever, such as Yunnan, Guangxi, Guangdong, Fujian, Zhejiang, Hainan and Taiwan. Moreover, the central area of this dengue outbreak was Qiyang County in Hunan, which is the closest geographical location in Hunan adjacent to Guangxi and Guangdong. This map was draw by authors according to the reference data

The geographic relationships between Hunan Province and the DENV outbreak areas in China were analysed. The results showed that Hunan, which is surrounded by Yunnan, Guangdong, Guangxi, Hainan, Fujian, Zhejiang and the other dengue outbreak areas, became a central area of the DENV epidemic (Fig. 1) The map in the figure was drawn by the authors of this study. Part of the data in the figure was cited from Zhao et al. [26], and part of the data was provided by local Centers for Disease Control and Prevention.

**Fig. 2:**
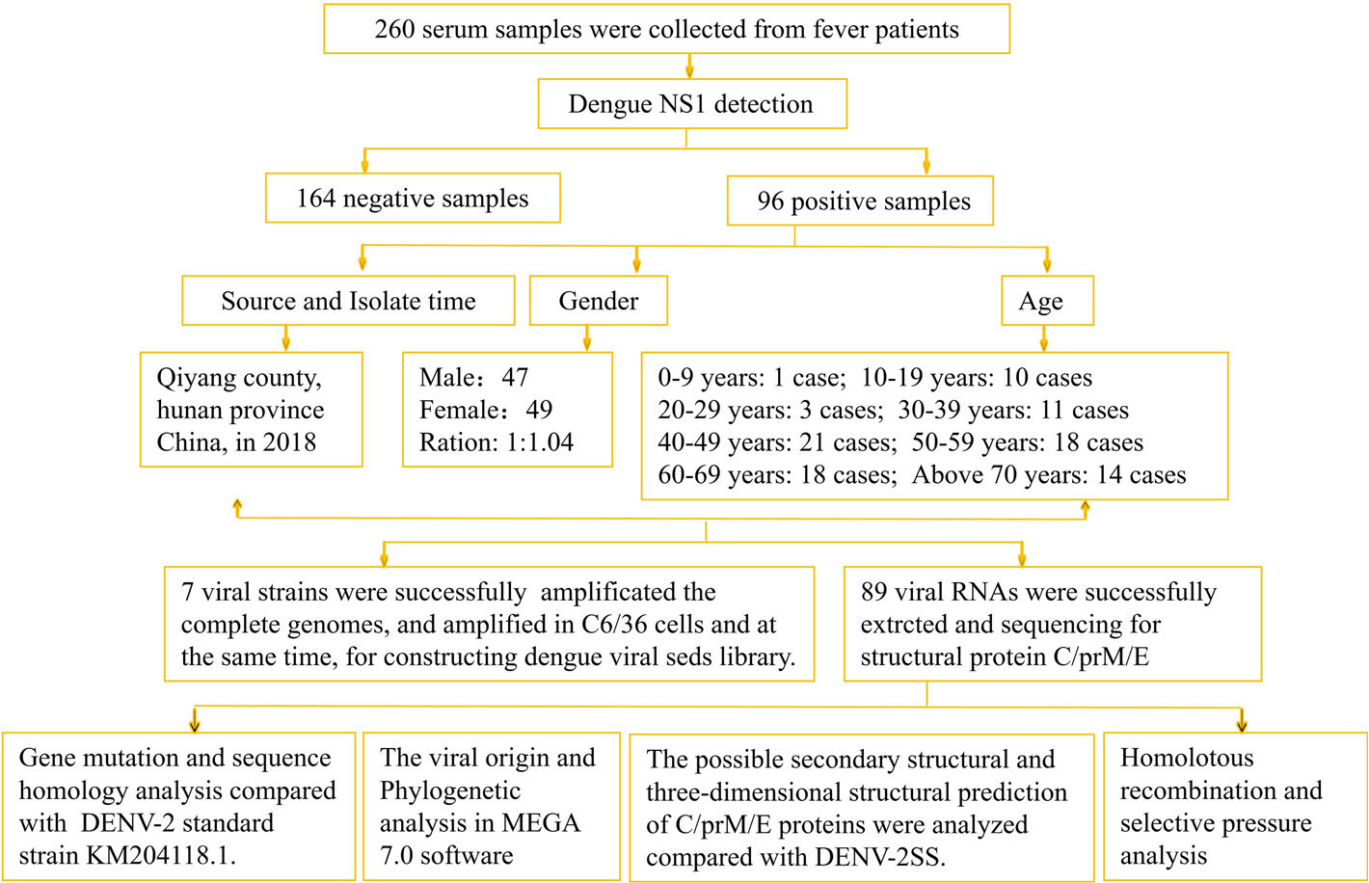
The study design and the characteristics of the study subjects. Two hundred and sixty dengue fever patients were recruited in our study; among them, 96 cases were identified as dengue NS1-positive. NS1-positive serum samples were collected for virus amplification and viral RNA extraction. Phylogenetic analysis was then conducted to characterize the origin of DENV in Qiyang, Hunan, during the 2018 outbreak

During the DENV outbreak in Qiyang County, Hunan, in September 2018, a total of 260 serum samples of fever patients were collected, and 96 cases were confirmed to be NS1-positive by colloidal gold testing. Seven strains were proliferated in C6/36 cells for over 6 days to build a viral seed pool of Hunan DENV. Eighty-nine viral RNA genomes were successfully extracted directly from the 96 NS1-positive serum samples, followed by gene sequencing of the DENV structural protein C/prM/E genes. Phylogenetic analysis, recombination and selection pressure analysis, and potential secondary structure prediction based on structural gene sequences originating from epidemic strains were performed to understand the genetic characterization, potential source, and evolution of the epidemic DENV strain. The study design is shown in Fig. 2.

### Phylogenetic analysis

**Fig. 3:**
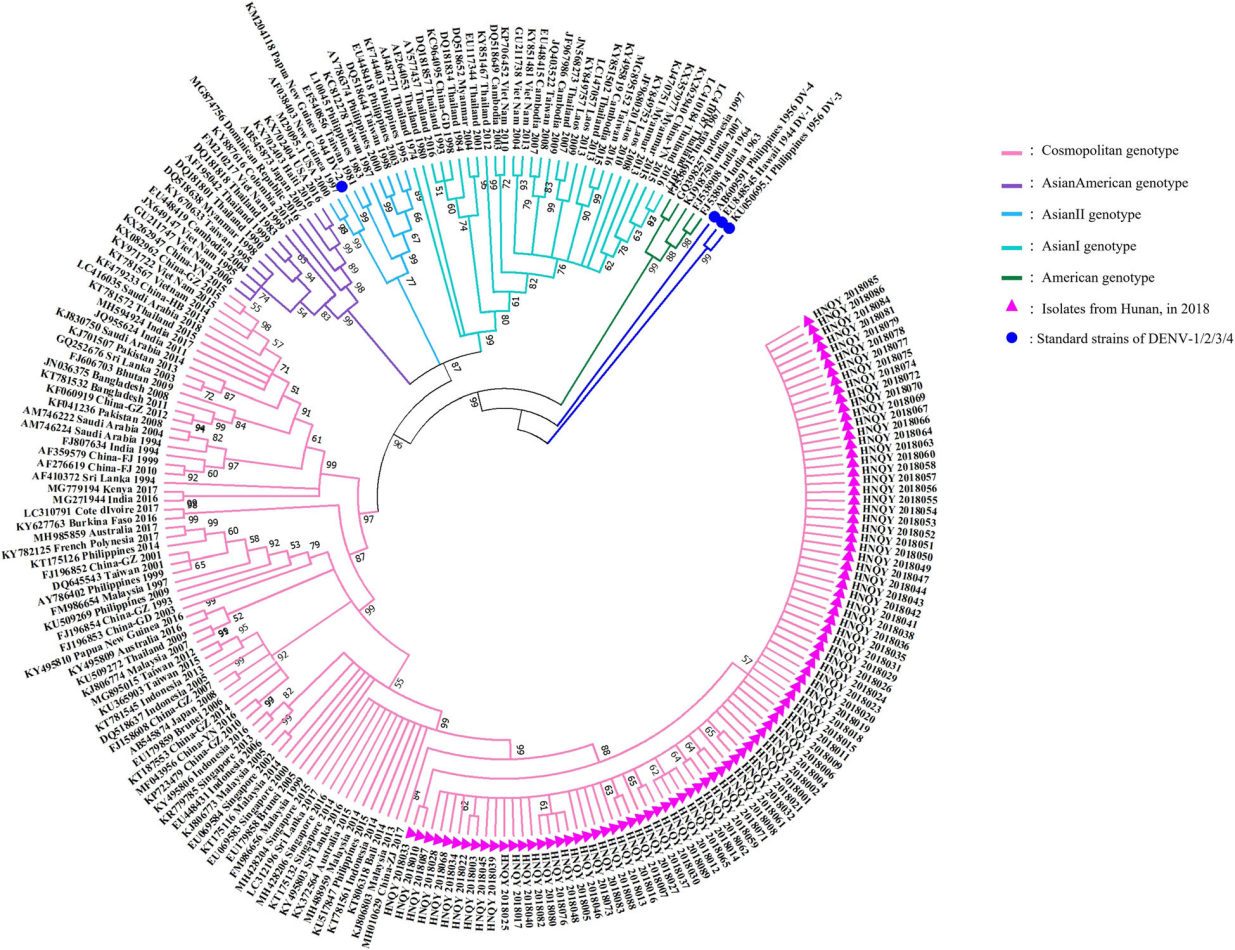
Phylogenetic tree of the E protein of DENV-2 epidemic strains in Hunan Province, China, in 2018. The phylogenetic trees were constructed by the maximum-likelihood method with a Kimura 2-parameter model using MEGA 7.0 software (https://www.megasoftware.net). The red triangles in the picture represent the 89 epidemic strains from Hunan, and the blue dots represent the standard strains (DENV-1/2/3/4)

The E protein gene sequences of 129 representative DENV-2 strains and four serotypes of standard strains were selected to construct phylogenetic trees with MEGA software version 7.0. The results showed that all 89 strains in this study were of the cosmopolitan DENV-2 genotype. The closest relative was the Zhejiang epidemic strain (MH010629, 2017), followed by strains isolated from Malaysia (KJ806803, 2013), Bali (KT806318, 2014), Indonesia (KT781561, 2014), and the Philippines (KU517847, 2015) (Fig. 3). Among the neighbouring provinces of Hunan Province, the provinces of Zhejiang, Yunnan and Guangdong each had more than 1000 reported cases of dengue fever in 2017, and all four serotypes were detected in each province [15, 20]. There were also reported cases in Fujian Province in 2017. Only one amino acid mutation (I431V/A) was observed in all 89 epidemic strains compared with the nearest related strain from Zhejiang (MH010629, 2017). These data suggest that the causative agent of the DENV outbreak in Hunan Province in 2018 may have come from the epidemic strains in Zhejiang Province in 2017.

### Bases and amino acid mutations

**Fig. 4:**
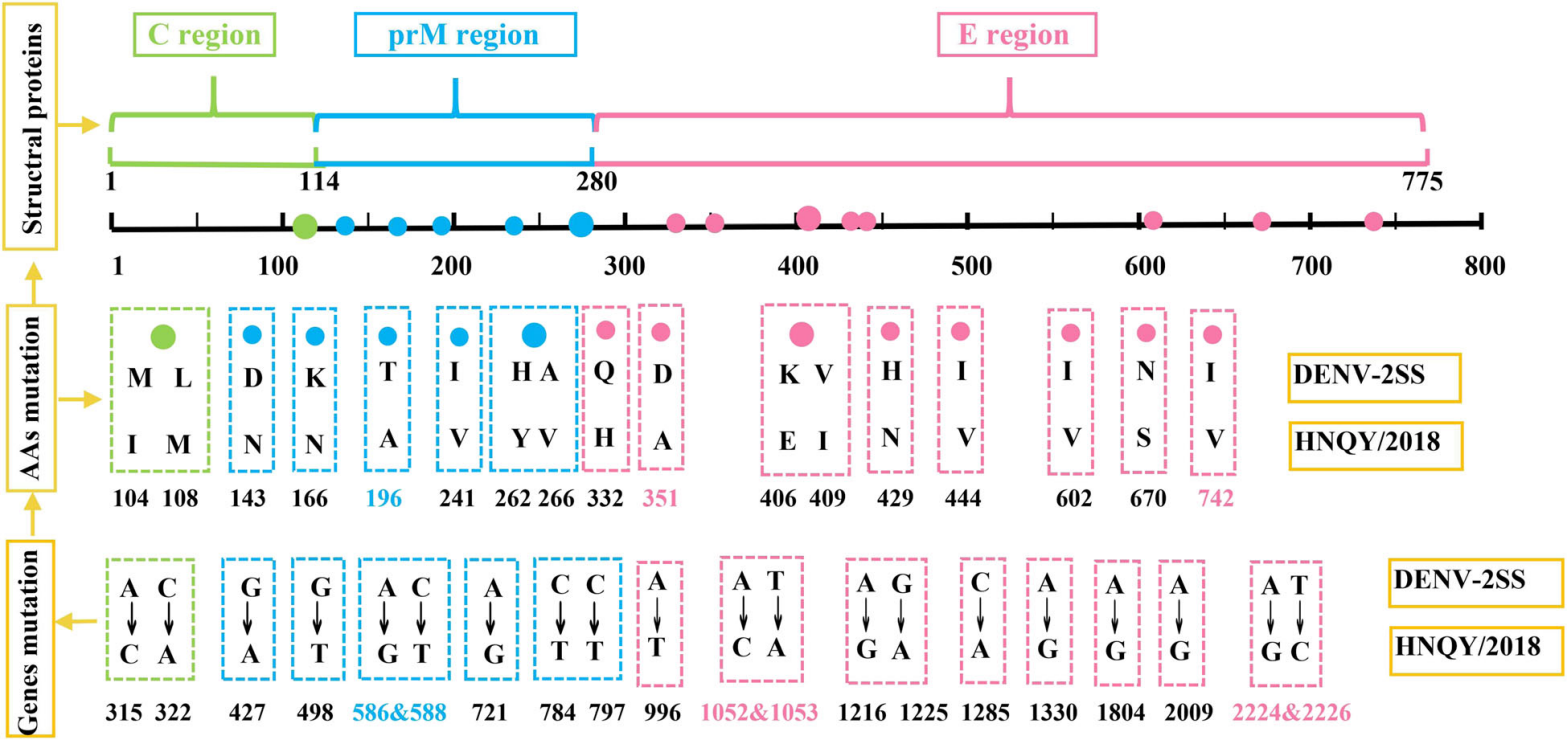
Map of the gene and amino acid mutation sites of the structural proteins from the epidemic strains from Hunan (HNQY2018001–2018089) compared to the DENV-2 standard strain KM204118.1

Three structural protein-overlapping fragments from epidemic strains were obtained by PCR amplification. After sequencing, the proteins were effectively spliced, and the length of the coding nucleotide sequences was 2325 nt, which encoded 775 amino acids. The homology between isolates was 99.7–100%, and the amino acid (AA) sequence of the E protein was highly conserved. By comparison, the homology of nucleotide and amino acid sequences between the 89 epidemic strains and DENV-2SS was 93.5 and 97.8%, respectively. Compared with DENV-2SS, the epidemic strains had two hundred fifteen mutated bases in the structural protein region, among which 195 were synonymous mutations and 20 were non-synonymous mutations, leading to 17 AA substitutions (Fig. 4). Two AA substitutions at the 104th (C104: M → I) and 108th (C108: L → M) amino acids were observed in protein C in the isolated strains; six amino acid mutations, including at the 143rd (M29: D → N), 166th (M52: K → N), 196th (M82: T → A), 241st (M127: I → V), 262nd (M148: H → Y), and 266th (M152: A → V) amino acids, occurred in the structural protein prM/M; and nine amino acid mutations, including at the 332nd (E52: Q → H), 351st (E71: D → A), 406th (E126: K → E), 409th (E129: V → I), 429th (E149: H → N), 444th (164: I → V), 602nd (E322: I → V), 670th (E390: N → S), and 742nd (E462: I → V) amino acids, were observed in structural protein E (Fig. 4).

### Potential secondary structure of the structural protein region

**Fig. 5:**
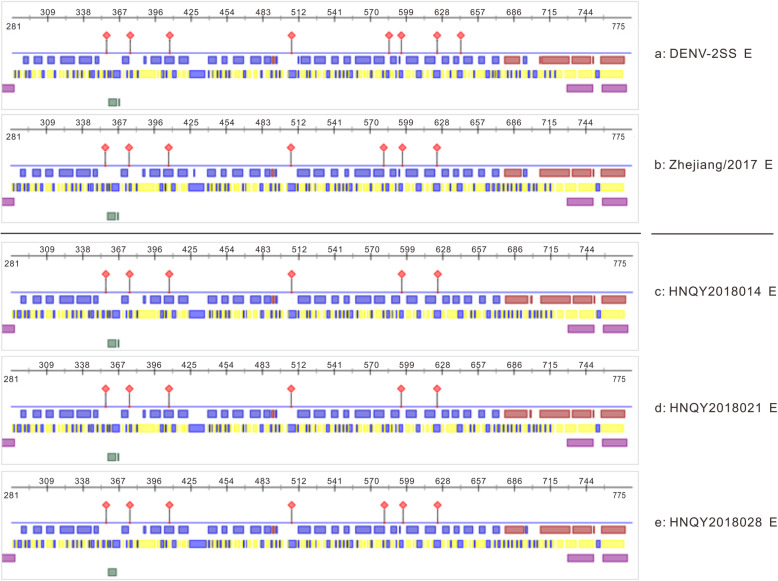
Protein secondary structure predictions for the Hunan epidemic strains, DENV-2SS (KM204118) and the Zhejiang/2017 (MG356770) strain. Note: The red rhombus denotes the protein binding region, the yellow dot denotes the DNA binding region, and the purple dot denotes the RNA binding region. Red and blue in the first line represent the helix and strand regions, respectively. Yellow and blue in the second line represent the buried and exposed regions, respectively. Purple and green in the third and fourth lines indicate the helical transmembrane and disordered regions, respectively

The protein secondary structures of the DENV-2 standard strain KM204118 and three randomly selected sequences (HNQY2018014, 021, and 028) from the 89 isolated strains were predicted. Compared with DENV-2SS, the Hunan epidemic strains lacked one nucleotide-binding site (site 6) and one DNA-binding site (site 18) as well as one protein binding region (sites 4 and 5) in the capsid protein (Fig. S1), while one new DNA binding site (site 74) and two new protein binding sites (sites 19 and 29) were observed in the isolated strains. Moreover, variations in the disordered region were found among the Hunan epidemic strains, DENV-2SS and the Zhejiang/2017 strain (Fig. S1). In the prM/M region, which contained 166 amino acids, the protein secondary structure of the epidemic strains was highly consistent with that of the Zhejiang/2017 strain (Fig. S2). However, compared to DENV-2SS, three protein binding regions were missing in the Hunan epidemic strains (sites 122, 133, and 220), and one novel protein binding region had emerged (site 144). Additionally, one helical transmembrane region of the isolates visibly differed from DENV-2SS, and eight significant changes were observed in the buried and exposed regions, while no noticeable variations were found in the strand or helix regions (Fig. S2). Three protein binding sites (sites 584, 596, and 642) were missing at the 495-AA locus of the E protein, one novel protein binding location (site 377) was observed in the Hunan isolates, four considerable alterations were also observed in the exposed and buried regions, and minor changes were found in the helical transmembrane and disordered region (Fig. 5). Moreover, there were 22 changes in strand regions. Of these, 11 were new (120, 166, 192, 309, 334, 347, 446, 455, 512, 582–584, 591), 11 were missing (101, 102, 124, 141, 207, 290, 294, 553, 607, 636, 651, 692–695), and nearly 70% of the changes occurred in E proteins. Nevertheless, compared with the Zhejiang 2017 strain, there was no significant change in the protein binding region or the polynucleotide binding region in the structural proteins (C, prM/M, and E) (Fig. 5, Fig. S1, and Fig. S2).

### Possible three-dimensional structure of the structural protein E genes

**Fig. 6:**
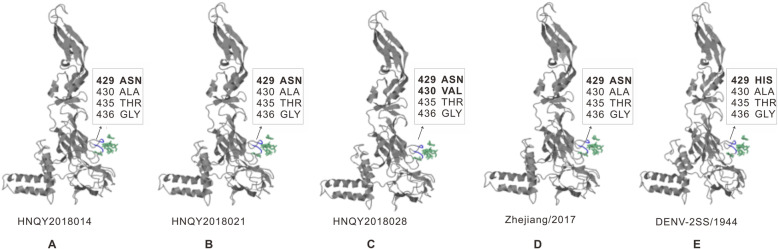
Predicted possible three-dimensional structures of the structural protein E genes of three representative 2018 Hunan epidemic strains (HNQY2018014, HNQY2018021, and HNQY2018028), the most closely related strain (Zhejiang/2017, MH010629), and the DENV-2 standard strain (DENV2-SS, KM204118). Blue indicates predicted protein binding sites. There are 4 possible binding sites in the E protein regions of these five strains

The possible three-dimensional structures of the structural proteins of the representative epidemic strains (HNQY2018014, 021, and 028) were predicted and compared with those of the DENV2-SS and Zhejiang/2017 strains. Homology modelling revealed that the five strains had the same three-dimensional structure. In addition, binding sites were predicted by the 3DLigandSite ligand binding site prediction server, and four protein binding sites (HIS429, ALA430, THR435, and GLY436) were observed in DENV-2SS (Fig. 6e). The Hunan epidemic strains and the Zhejiang/2017 strain had the same binding sites at ASN429, THR435, and GLY436 (Fig. 6d). HNQY2018028 had two different binding sites (429 and 430) compared to DENV-2SS (Fig. 6) and one different binding site (429) compared to the Zhejiang/2017 strain.

### Recombination and selection pressure analysis

RDP4 software was used to analyse potential recombination events among HNQY2018001–HNQY2018089 and other representative DENV-2 virus strains. Preliminary analysis results showed that no recombination event occurred in these DENV-2 strains (*p* < 0.05). The structural proteins of 200 strains were analysed, including 111 representative strains of DENV-2 and the 89 isolated strains. The MEEM method identified the maximum number of actively selected sites (*n* = 16). However, the FEL, IFEL and FUBAR methods indicated that all 775 sites were under negative pressure (Table 1). Therefore, no significant evidence of positive selection was found with at least three different methods, so positive selection pressure at these sites cannot be determined.

**Table 1:** Selection pressure analysis of the structural protein of DENV-2 (*n* = 202) using FEL, IFEL, MEME, and FUBAR

Serial number	AA position	FEL		IFEL		MEME		FUBAR	
		ω	p-Value	ω	p-Value	ω	p-Value	ω	Posterior Prob(α<β)
1	11	/	/	/	/	>100	0.083	/	/
2	19	/	/	/	/	>100	0.027	/	/
3	35	/	/	/	/	>100	0.066	0.132	0.963
4	207	/	/	/	/	>100	0.061	0.174	0.916
5	209	0.049	0.000	0.114	0.012	>100	0.078	0.068	1.000
6	228	/	/	/	/	>100	0.056	/	/
7	332	/	/	/	/	>100	0.032	/	/
8	400	/	/	/	/	>100	0.004	/	/
9	451	0.070	0.014	0.000	0.011	>100	0.069	0.082	0.998
10	463	/	/	/	/	>100	0.037	/	/
11	464	0.164	0.008	0.000	0.001	>100	0.000	0.182	0.988
12	474	/	/	/	/	>100	0.089	/	/
13	475	0.153	0.078	0.000	0.047	>100	0.053	0.130	0.983
14	488	/	/	/	/	>100	0.053	0.164	0.928
15	506	/	/	/	/	>100	0.052	/	/
16	521	/	/	/	/	>100	0.053	0.133	0.976

Note: Criteria to consider sites with significant evidence of positive selection: p-value < 0.1 in FEL, IFEL, and MEME, Posterior Prob (α < β) > 0.9 in FUBAR, and omega should be greater than 1. Sites that were found to be positive by at least one method are included in the list. “/”: represents that the site was not selected by the corresponding method as a positive or negative selection. “AA”: represents amino acids

## Discussion

In mainland China, dengue fever mainly occurs in Guangdong, Hainan, Zhejiang, Fujian, Guangxi, and other coastal regions or in Yunnan Province and Southeast Asian countries adjacent to Yunnan Province. Only scattered cases have been reported in inland China, but no large-scale dengue epidemic has been reported in the inland area to date. Hunan is an inland province of China located near 30 degrees north latitude. The climate is warm and humid from June to November, which provides a natural environment for the breeding of *Aedes albopictus*. Hunan Province is located near Guangdong, Guangxi, Zhejiang, and other areas with a high incidence of dengue fever. The total number of dengue infections in China was 5106 in 2018, which included 3250 cases in Guangdong Province, 217 cases in Zhejiang, and 172 cases in Hunan. This was the first dengue outbreak in Hunan, an inland province of China, which provides us with an early warning that dengue fever has gradually spread from coastal and border areas to inland areas of China and highlights the urgent need to monitor the cross-border and cross-regional transmission of dengue viruses.

In this study, we collected serum from 260 patients with dengue fever in Qiyang County, Hunan Province, and 96 of the cases were confirmed to be NS1-positive. Of them, 89 viral RNAs were extracted, and structural protein gene fragments (HNQY2018001–089) were obtained by amplification of overlapping fragments with a length of 2325 nucleotides. Phylogenetic tree analysis showed that all isolated strains were of the cosmopolitan DENV-2 genotype, belonged to one cluster of the ML tree and were closely related to the Zhejiang strain (2017, MH110588). Additionally, the isolated strains were closely related to strains from Malaysia (KJ806803, 2013), Bali (KT806318, 2014), Indonesia (KT781561, 2014) and the Philippines (KU517847, 2015). Although all four serotypes of dengue virus were prevalent in Zhejiang Province in 2017, the vast majority of the epidemic strains were still of the cosmopolitan DENV-2 genotype, and the encoded protein of this epidemic strain has only one amino acid that differs from that of the epidemic strain in Hunan Province in 2018. This result suggested that the DENV-2 epidemic in Hunan was possibly imported from Southeast Asian countries, such as Malaysia, Indonesia or the Philippines, passed through Zhejiang Province, and then spread to Hunan Province.

Compared with that of the standard strains, 17 amino acid substitutions were observed in the structural protein C/prM/E of all 89 epidemic strains. The prM-E protein is the main structural protein of flaviviruses and is related to virulence, host affinity, virus adsorption, penetration, and cell fusion [27]. Hydrophobic amino acids play an important role in maintaining the tertiary structure of proteins due to their hydrophobic interactions and may impact the virulence of the virus. Tamm et al. found that hydrophobic domains affect the virulence potential of Yersinia enterocolitica [28]. Sainz et al. determined that single hydrophobic amino acids play an important role in transcriptional activation in vivo [29]. In our study, three hydrophobic amino acids in the CDS region were mutated into hydrophilic amino acids, namely, the 196th (M82: T → A), 262nd (M148: H → Y), and 351st (E71: D → A) amino acids. In addition, a neutral amino acid became a basic amino acid at the 332nd (E52: Q → H) position, and two positively charged amino acids were converted into negatively charged amino acids at the 406th (E126: K → E) and 429th (E139: H → N) positions. Mutations in these amino acids have not been reported, and changes in polarity or charge of amino acids may affect the functions of the prM and E proteins; however, further studies are needed to confirm these hypotheses. DENV E protein domain III (E295 ~ E395) undergoes immunoglobulin G (IgG)-like folding and plays an important role in mediating the fusion of viruses and host receptors [30]. In this study, there were two amino acid changes in the EDIII domain at the 602nd (E322: I → V) and 670th (E390: N → S) positions. It has been reported that the mutation of E390 from asparagine to serine can enhance the replication ability of viruses [31], but the influence of the E322 amino acid mutation remains to be determined.

Changes in protein secondary structure will affect enzyme activity. Compared with the DENV-2 standard strain (DENV2-SS), the isolated strains lacked eight protein binding sites (4, 5, 122, 120, 133, 584, 596, and 642) and two polynucleotide binding sites (6 and 18). Moreover, four new protein binding sites (19, 29, 144, and 377) and one polynucleotide binding site (74) emerged in the isolated strains. Furthermore, approximately eight obvious changes were observed in the buried and exposed regions. All of the above changes may lead to the diversification of protein structural domains and further influence protein function. Homologous modelling and prediction of the possible 3D structures of structural proteins showed that the structural proteins from the epidemic strains and DENV2-SS had similar 3D structures and shared 4 predicted protein binding sites, and only one protein binding site (429th) differed between them (DENV2-SS: HIS429; Zhejiang/2017 and Hunan epidemic strain: ASN429).

The analysis showed that there were no recombination events between the Hunan epidemic strains and the 111 DENV-2 reference strains, and no distinct positive selection sites were detected in the structural proteins, which contained 775 amino acids, which suggests that these structural protein-coding genes were conserved.

## Conclusions

This study described the characteristics of the structural protein coding sequences in DENV-2 originating from the 2018 outbreak in Hunan Province in inland China. This will benefit follow-up studies of DENV in China and Southeast Asia. Our findings also indicate that the transmission region of DENV has gradually spread from China’s border and coastal areas to inland China. These findings provide us with a warning that the dengue fever epidemic in China is becoming increasingly serious and difficult to control and emphasizes the urgent need to monitor the cross-border spread of DENV.

## Supplementary Information

**Additional file 1: Table S1.** Primers for serotype identification of dengue virus.

**Additional file 2: Table S2.** Primers for amplification of the structural protein (C/prM/E) of DENV-2.

**Additional file 3: Table S3.** Reference sequences for phylogenetic analysis.

**Additional file 4: Table S4.** Reference sequences of DENV-2 for recombination and selection pressure analysis.

**Additional file 5: Figure S1.** Secondary structure prediction of capsid protein of DENV-2SS, Zhejiang/2017 and HNQY (2,018,014, 2,018,021 and 2,018,028).

**Additional file 6: Figure S2.** Secondary structure prediction of prM protein of DENV-2SS, Zhejiang/2017 and HNQY (2,018,014, 2,018,021 and 2,018,028).

## Data Availability

All the data supporting our findings are contained within the manuscript. The structural protein gene sequences of the 89 epidemic strains in Hunan Province in 2018 were uploaded to the National Center for Biotechnology Information (NCBI) GenBank® database (http://www.ncbi.nim.nih.gov/GenBank/index.html) (GenBank IDs: MK543451–MK543470, MK543472–MK543478, MK543480–MK543492, and MK949396–MK949438).

## Abbreviations

AA: Amino acid
CCDC: Chinese Center for Disease Control
DENV: Dengue virus
DENV-1/2/3/4: Dengue virus serotype 1/2/3/4
DF: Dengue fever
DENV-2SS: Dengue virus serotype 2 standard strain
FEL: Fixed Effect Likelihood
FUBAR: Fast, Unconstrained Bayesian AppRoximation
GARD: Genetic Algorithm Recombination Detection
IFEL: Internal Fixed Effect Likelihood
IgG: Immunoglobulin G
MEEM: Mixed Effect Evolution Model
MEGA: Molecular Evolutionary Genetics Analysis
NCBI: National Center for Biotechnology Information
NS1: Non-structural protein 1
NT: Nucleotides
PCR: Polymerase chain reaction
WHO: World Health Organization
