# Supplementary material for: Molecular characterization of the viral structural protein genes in the first outbreak of dengue virus type 2 in Hunan Province, inland China in 2018
Source: BMC Infect Dis. 2021 Feb 10;21:166. doi: 10.1186/s12879-021-05823-3 (PMC7874035; doi:10.1186/s12879-021-05823-3)
Supplement: Supplementary file 1 — Additional file 1: Table S1. Primers for serotype identification of dengue virus. [file 12879_2021_5823_MOESM1_ESM.doc]

Table S1 Primers for serotype identification of dengue virus

| Serial number | Primer name | Sequence (5’→3’) | Size (bp) |
| --- | --- | --- | --- |
| 1 | DV--F | TCAATATGCTGAAACGCGCGAGAAACCG | 511 |
|  | DV-R | TTGCACCAACAGTCAATGTCTTCAGGTTC |  |
| 2 | DV1-F | TCAATATGCTGAAACGCGCGAGAAACCG | 482 |
|  | DV1-R | CGTCTCAGTGATCCGGGGG |  |
| 3 | DV2-F | TCAATATGCTGAAACGCGCGAGAAACCG | 119 |
|  | DV2-R | CGCCACAAGGGCCATGAACAG |  |
| 4 | DV3-F | TCAATATGCTGAAACGCGCGAGAAACCG | 290 |
|  | DV3-R | CGCCACAAGGGCCATGAACAG |  |
| 5 | DV4-F | TCAATATGCTGAAACGCGCGAGAAACCG | 392 |
|  | DV4-R | CTCTGTTGTCTTAAACAAGAGA |  |
