# Supplementary material for: Molecular characterization of the viral structural protein genes in the first outbreak of dengue virus type 2 in Hunan Province, inland China in 2018
Source: BMC Infect Dis. 2021 Feb 10;21:166. doi: 10.1186/s12879-021-05823-3 (PMC7874035; doi:10.1186/s12879-021-05823-3)
Supplement: Supplementary file 2 — Additional file 2: Table S2. Primers for amplification of the structural protein (C/prM/E) of DENV-2. [file 12879_2021_5823_MOESM2_ESM.doc]

Table S2 Primers for amplification of the structural protein (C/prM/E) of DENV-2

| Serial number | Primer name | Sequence (5’→3’) | Size (bp) |
| --- | --- | --- | --- |
| 1 | F1 | AGTCTACGTGGACCGACAAAG | 394 |
|  | R1 | TGTCCTCTGCGTCTTGACG |  |
| 2 | F2 | TGCTGAAACGCGAGAGAAACC | 1380 |
|  | R2 | TTGCCCGGAGCTGAAGTTAC |  |
| 3 | F3 | TGCAGTCGGAAATGACACAGG | 1335 |
|  | R3 | TGTAGTTTCCTTAGTACGTCCG |  |
