# Supplementary material for: Molecular characterization of the viral structural protein genes in the first outbreak of dengue virus type 2 in Hunan Province, inland China in 2018
Source: BMC Infect Dis. 2021 Feb 10;21:166. doi: 10.1186/s12879-021-05823-3 (PMC7874035; doi:10.1186/s12879-021-05823-3)
Supplement: Supplementary file 3 — Additional file 3: Table S3. Reference sequences for phylogenetic analysis. [file 12879_2021_5823_MOESM3_ESM.doc]

Table S3 Reference sequences for **phylogenetic analysis**

| Serial number | Accession number | Country | Year | Type/subtype |
| --- | --- | --- | --- | --- |
| 1 | KX372564 | Australia | 2015 | 2-Cosmopolitan |
| 2 | KY495809 | Australia | 2016 | 2-Cosmopolitan |
| 3 | MH985859 | Australia | 2017 | 2-Cosmopolitan |
| 4 | KT806318 | Bali | 2014 | 2-Cosmopolitan |
| 5 | JN036375 | Bangladesh | 2008 | 2-Cosmopolitan |
| 6 | KT781532 | Bangladesh | 2011 | 2-Cosmopolitan |
| 7 | FJ606703 | Bhutan | 2009 | 2-Cosmopolitan |
| 8 | EU179858 | Brunei | 2005 | 2-Cosmopolitan |
| 9 | EU179859 | Brunei | 2006 | 2-Cosmopolitan |
| 10 | KY627763 | Burkina Faso | 2016 | 2-Cosmopolitan |
| 11 | DQ518649 | Cambodia | 2003 | 2-Asian I |
| 12 | EU448419 | Cambodia | 2004 | 2-Asian Ameirican |
| 13 | EU448415 | Cambodia | 2007 | 2-Asian I |
| 14 | JF967986 | Cambodia | 2009 | 2-Asian I |
| 15 | KY495819 | Cambodia | 2015 | 2-Asian I |
| 16 | FJ196854 | China-GZ | 1993 | 2-Cosmopolitan |
| 17 | KC964095 | China-GD | 1998 | 2-Asian I |
| 18 | AF359579 | China-FJ | 1999 | 2-Cosmopolitan |
| 19 | FJ196852 | China-GZ | 2001 | 2-Cosmopolitan |
| 20 | FJ196853 | China-GD | 2003 | 2-Cosmopolitan |
| 21 | FJ158608 | China-GZ | 2007 | 2-Cosmopolitan |
| 22 | KP723479 | China-GD | 2010 | 2-Cosmopolitan |
| 23 | AF276619 | China-FJ | 2010 | 2-Cosmopolitan |
| 24 | KF606919 | China-GZ | 2012 | 2-Cosmopolitan |
| 25 | KF479233 | China-HB | 2013 | 2-Cosmopolitan |
| 26 | KT187553 | China-GD | 2014 | 2-Cosmopolitan |
| 27 | KX082962 | China-GZ | 2015 | 2-Cosmopolitan |
| 28 | KX262940 | China-YN | 2015 | 2-Asian I |
| 29 | KX262947 | China-YN | 2015 | 2-Cosmopolitan |
| 30 | MF043956 | China-YN | 2016 | 2-Cosmopolitan |
| 31 | MG356770 | China-ZJ | 2017 | 2-Cosmopolitan |
| 32 | KY887616 | Colombia | 2015 | 2-Asian Ameirican |
| 33 | LC310791 | Cote dlvoire | 2017 | 2-Cosmopolitan |
| 34 | MG874756 | Dominican Republic | 2016 | 2-Asian Ameirican |
| 35 | KY782125 | French Polynesia | 2017 | 2-Cosmopolitan |
| 36 | KX702403 | Haiti | 2016 | 2-Asian Ameirican |
| 37 | FJ538914 | India | 1963 | 2-Ameirican |
| 38 | FJ538908 | India | 1964 | 2-Ameirican |
| 39 | FJ538910 | India | 1967 | 2-Ameirican |
| 40 | FJ807634 | India | 1994 | 2-Cosmopolitan |
| 41 | KJ918750 | India | 2007 | 2-Ameirican |
| 42 | JQ955624 | India | 2011 | 2-Cosmopolitan |
| 43 | MG271944 | India | 2016 | 2-Cosmopolitan |
| 44 | MH594924 | India | 2017 | 2-Cosmopolitan |
| 45 | GQ398257 | Indonesia | 1997 | 2-Ameirican |
| 46 | DQ518637 | Indonesia | 2005 | 2-Cosmopolitan |
| 47 | EU448431 | Indonesia | 2006 | 2-Cosmopolitan |
| 48 | KT781545 | Indonesia | 2013 | 2-Cosmopolitan |
| 49 | KT781561 | Indonesia | 2014 | 2-Cosmopolitan |
| 50 | KY495806 | Indonesia | 2016 | 2-Cosmopolitan |
| 51 | AB545873 | Japan | 2007 | 2-Asian Ameirican |
| 52 | AB545874 | Japan | 2008 | 2-Cosmopolitan |
| 53 | MG779194 | Keyya | 2017 | 2-Cosmopolitan |
| 54 | KY849752 | Laos | 2008 | 2-Asian I |
| 55 | KY849757 | Laos | 2009 | 2-Asian I |
| 56 | JF968020 | Laos | 2010 | 2-Asian I |
| 57 | LC147057 | Laos | 2013 | 2-Asian I |
| 58 | FM986654 | Malaysia | 1997 | 2-Cosmopolitan |
| 59 | FM986656 | Malaysia | 1999 | 2-Cosmopolitan |
| 60 | KJ806773 | Malaysia | 2005 | 2-Cosmopolitan |
| 61 | KJ806774 | Malaysia | 2007 | 2-Cosmopolitan |
| 62 | KJ806803 | Malaysia | 2013 | 2-Cosmopolitan |
| 63 | KT175116 | Malaysia | 2014 | 2-Cosmopolitan |
| 64 | MH488959 | Malaysia | 2014 | 2-Cosmopolitan |
| 65 | DQ518638 | Myanmar | 1998 | 2-Asian Ameirican |
| 66 | DQ518652 | Myanmar | 2004 | 2-Asian I |
| 67 | KJ470751 | Myanmar | 2013 | 2-Asian I |
| 68 | KX357977 | Myanmar | 2015 | 2-Asian I |
| 69 | KF041236 | Pakistan | 2008 | 2-Cosmopolitan |
| 70 | KJ701507 | Pakistan | 2013 | 2-Cosmopolitan |
| 71 | KY495810 | Papua New Guinea | 2016 | 2-Cosmopolitan |
| 72 | L10045 | Philippines | 1983 | 2-Asian II |
| 73 | KF744403 | Philippines | 1995 | 2-Asian II |
| 74 | AY786402 | Philippines | 1999 | 2-Cosmopolitan |
| 75 | AY786374 | Philippines | 2000 | 2-Asian II |
| 76 | EU448418 | Philippines | 2003 | 2-Asian II |
| 77 | KU509269 | Philippines | 2009 | 2-Cosmopolitan |
| 78 | KT175126 | Philippines | 2014 | 2-Cosmopolitan |
| 79 | KU517847 | Philippines | 2015 | 2-Cosmopolitan |
| 80 | AM746224 | Saudi Arabia | 1994 | 2-Cosmopolitan |
| 81 | AM746222 | Saudi Arabia | 2004 | 2-Cosmopolitan |
| 82 | KJ830750 | Saudi Arabia | 2014 | 2-Cosmopolitan |
| 83 | LC416035 | Saudi Arabia | 2018 | 2-Cosmopolitan |
| 84 | EU069583 | Singapore | 2000 | 2-Cosmopolitan |
| 85 | EU069584 | Singapore | 2002 | 2-Cosmopolitan |
| 86 | KR779785 | Singapore | 2013 | 2-Cosmopolitan |
| 87 | KT175132 | Singapore | 2014 | 2-Cosmopolitan |
| 88 | MH428204 | Singapore | 2015 | 2-Cosmopolitan |
| 89 | MH428206 | Singapore | 2016 | 2-Cosmopolitan |
| 90 | AF410372 | Sri Lanka | 1994 | 2-Cosmopolitan |
| 91 | GQ252676 | Sri Lanka | 2003 | 2-Cosmopolitan |
| 92 | KY495803 | Sri Lanka | 2016 | 2-Cosmopolitan |
| 93 | LC312196 | Sri Lanka | 2017 | 2-Cosmopolitan |
| 94 | EF540856 | Taiwan | 1981 | 2-Asian II |
| 95 | KC812278 | Taiwan | 1987 | 2-Asian II |
| 96 | KY670633 | Taiwan | 1995 | 2-Asian Ameirican |
| 97 | DQ518644 | Taiwan | 1998 | 2-Asian II |
| 98 | DQ645543 | Taiwan | 2001 | 2-Cosmopolitan |
| 99 | JQ403522 | Taiwan | 2008 | 2-Asian I |
| 100 | MG895015 | Taiwan | 2012 | 2-Cosmopolitan |
| 101 | KU365903 | Taiwan | 2015 | 2-Cosmopolitan |
| 102 | MG895152 | Taiwan | 2016 | 2-Asian I |
| 103 | AJ487271 | Thailand | 1974 | 2-Asian I |
| 104 | AF264053 | Thailand | 1980 | 2-Asian I |
| 105 | DQ181818 | Thailand | 1983 | 2-Asian Ameirican |
| 106 | DQ181834 | Thailand | 1984 | 2-Asian I |
| 107 | DQ181801 | Thailand | 1990 | 2-Asian Ameirican |
| 108 | DQ181857 | Thailand | 1993 | 2-Asian I |
| 109 | AF195042 | Thailand | 1999 | 2-Asian Ameirican |
| 110 | EU117344 | Thailand | 2001 | 2-Asian I |
| 111 | JN568273 | Thailand | 2007 | 2-Asian I |
| 112 | KU509272 | Thailand | 2009 | 2-Cosmopolitan |
| 113 | KY851467 | Thailand | 2012 | 2-Asian I |
| 114 | KY851502 | Thailand | 2013 | 2-Asian I |
| 115 | KT781572 | Thailand | 2015 | 2-Cosmopolitan |
| 116 | AY577437 | Thailand | 2016 | 2-Asian I |
| 117 | LC410184 | Thailand | 2016 | 2-Asian I |
| 118 | LC410187 | Thailand | 2017 | 2-Asian I |
| 119 | M29095.1 | USA | 2000 | DV-2 |
| 120 | KX702404 | USA | 2016 | 2-Asian Ameirican |
| 121 | JX649147 | Viet Nam | 1995 | 2-Asian Ameirican |
| 122 | FM210217 | Viet Nam | 1999 | 2-Asian Ameirican |
| 123 | GU211738 | Viet Nam | 2004 | 2-Asian I |
| 124 | GU211747 | Viet Nam | 2006 | 2-Asian Ameirican |
| 125 | KP706452 | Viet Nam | 2010 | 2-Asian I |
| 126 | KY851481 | Viet Nam | 2013 | 2-Asian I |
| 127 | KT781567 | Viet Nam | 2014 | 2-Cosmopolitan |
| 128 | KY971722 | Viet Nam | 2015 | 2-Cosmopolitan |
| 129 | EU848545.1 | Hawaii | 1944 | DV-1 |
| 130 | KM204118 | New Guinea | 1944 | DV-2 |
| 131 | KU050695 | Philippines | 1956 | DV-3 |
| 132 | AB609591 | Philippines | 1956 | DV-4 |
