# Supplementary figures and images for: Molecular characterization of the viral structural protein genes in the first outbreak of dengue virus type 2 in Hunan Province, inland China in 2018
Source: BMC Infect Dis. 2021 Feb 10;21:166. doi: 10.1186/s12879-021-05823-3 (PMC7874035; doi:10.1186/s12879-021-05823-3)

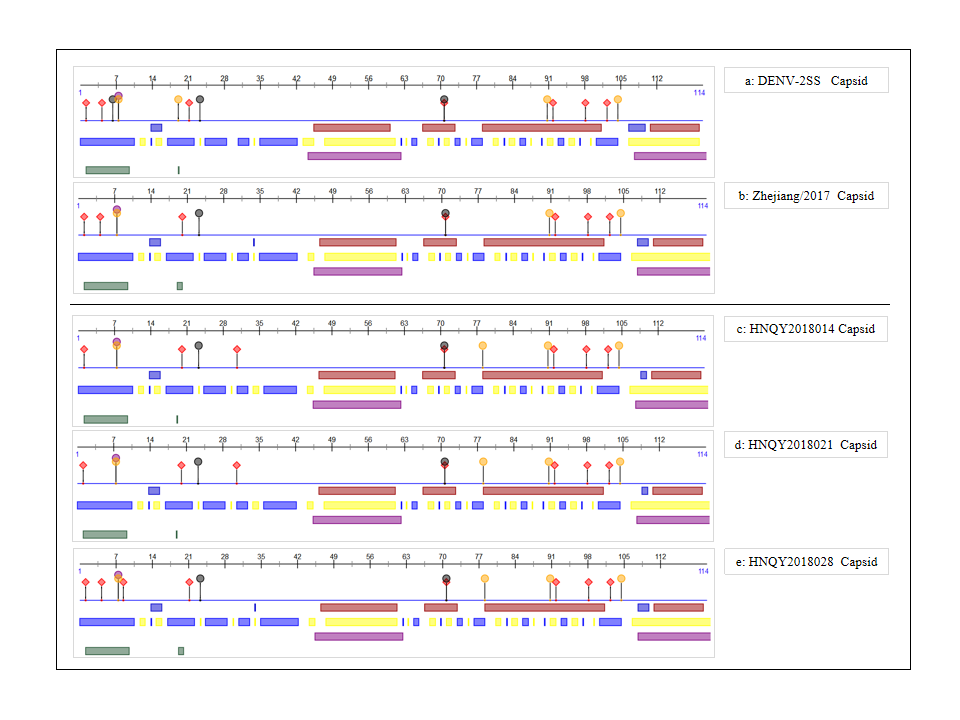


Figure S1. Secondary structure prediction of capsid protein of DENV-2SS, Zhejiang/2017 and HNQY (2018014, 2018021 and 2018028).

Supplement: Supplementary file 5 — Additional file 5: Figure S1. Secondary structure prediction of capsid protein of DENV-2SS, Zhejiang/2017 and HNQY (2,018,014, 2,018,021 and 2,018,028). [file 12879_2021_5823_MOESM5_ESM.doc]

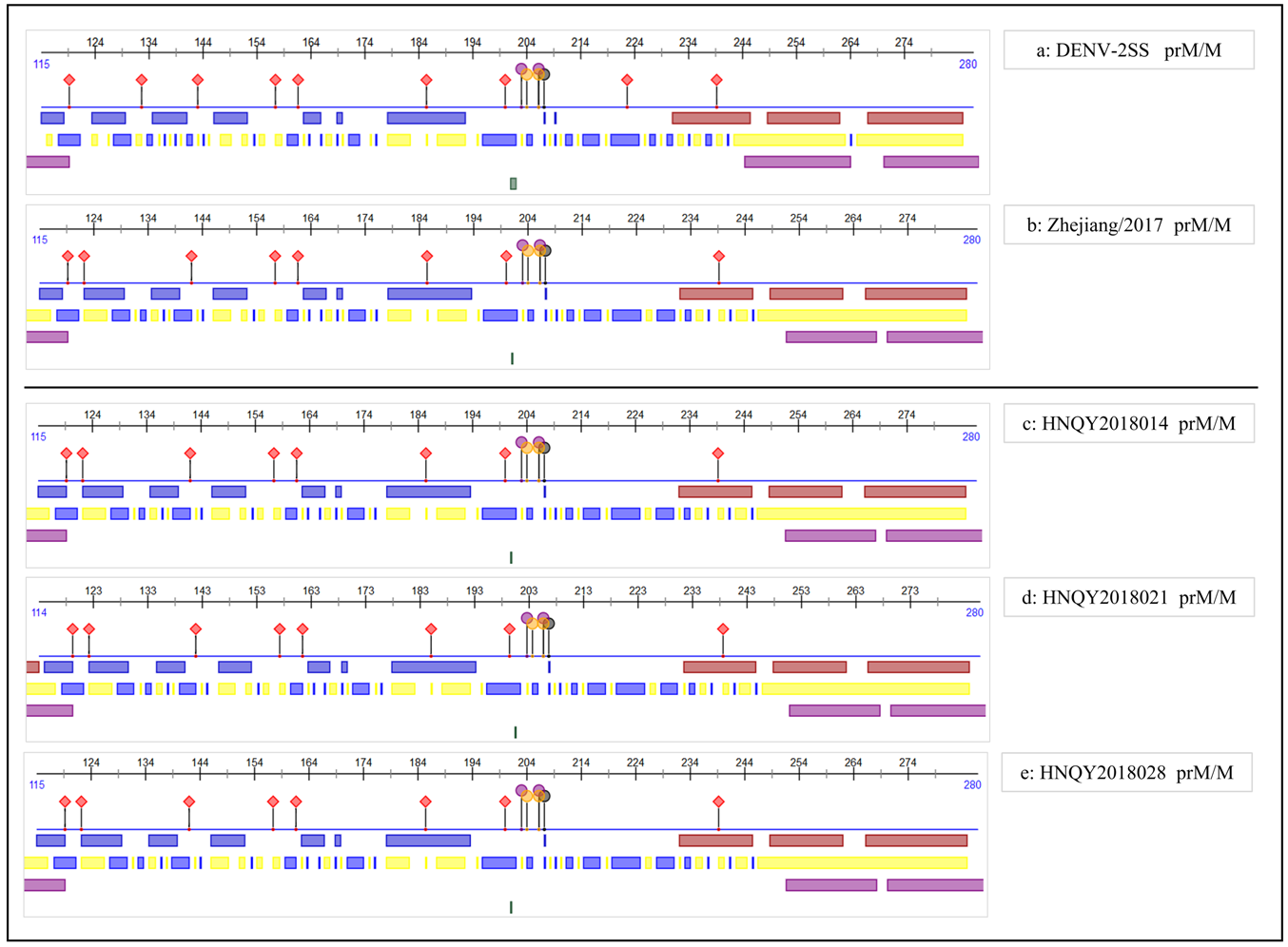


Figure S2. Secondary structure prediction of prM protein of DENV-2SS, Zhejiang/2017 and HNQY (2018014, 2018021 and 2018028).

Supplement: Supplementary file 6 — Additional file 6: Figure S2. Secondary structure prediction of prM protein of DENV-2SS, Zhejiang/2017 and HNQY (2,018,014, 2,018,021 and 2,018,028). [file 12879_2021_5823_MOESM6_ESM.doc]
